# Editorial: Neural Mechanisms Underlying Movement-Based Embodied Contemplative Practices

**DOI:** 10.3389/fnhum.2016.00169

**Published:** 2016-04-26

**Authors:** Laura Schmalzl, Catherine E. Kerr

**Affiliations:** ^1^Department of Family Medicine and Public Health, University of California San DiegoLa Jolla, CA, USA; ^2^VA San Diego Healthcare SystemLa Jolla, CA, USA; ^3^Department of Family Medicine, Brown UniversityProvidence, RI, USA

**Keywords:** yoga, tai chi, Feldenkrais, dance, somatics, embodiment, movement, contemplation

## Introduction

Compared to the extensive body of neuroscientific work on seated meditation practices, far fewer studies have investigated the neural mechanisms underlying movement-based contemplative practices such as yoga or tai chi. One likely reason is the inherent challenge of dealing with their multifaceted nature, typically involving specific movement sequences, regulation of the breath, and modulation of attention. Movement-based practices have, however, been found to be effective for relieving the symptoms of clinical conditions as diverse as cancer, Parkinson's disease (PD), chronic pain, fibromyalgia, post-traumatic stress disorder (PTSD), attention deficit hyperactivity disorder (ADHD), depression, and anxiety-related disorders. In addition, they have been shown to elicit measurable changes in physiological stress parameters, cognitive, and physical functioning as well experienced emotional states in healthy individuals. An important challenge for contemplative science is therefore to advance our understanding of the neurophysiological and neurocognitive mechanisms underlying these observed effects. The current Research Topic aims to make a contribution in this regard by outlining the state of the art of research on movement-based practices including yoga, tai chi, the Feldenkrais Method, as well as dance. The featured articles present empirical data, propose novel theoretical frameworks, and address the clinical implications of research within the field.

## Contributions to the current research topic

### Movement-based embodied contemplative practices

The first section of this Research Topic features perspective as well as hypothesis and theory articles addressing general aspects of movement-based contemplative approaches. Schmalzl et al. present an introductory perspective article defining the concept of movement-based embodied contemplative practices (MECPs). Drawing from examples of various movement-based practices and modern somatic therapeutic techniques, the authors explore how they are grounded in the concepts of embodiment, movement and contemplation. The practices are viewed through the lens of an enactive approach to cognition, which postulates that mental functions cannot be fully understood without reference to the physical body as well as the environment in which they are experienced. Russell and Arcuri discuss clinical and research implications of mindful movement. Specifically, the authors address how regulation of attention and working memory relate to mind-wandering in the context of both mindfulness and movement training. In addition, they propose that for some clinical populations mindful movement may be more suited than traditional seated mindfulness techniques. Clark et al. examine the relationship between motor and cognitive functioning, suggesting that mindful movement approaches are of particular relevance for developmental disorders such as ADHD. The authors propose a model of skilled attention in which motor plans, attention, and executive function are seen as mutually co-defining aspects of skilled behavior, and discuss direct clinical implications of their theoretical framework. Lastly, Payne and Crane-Godreau delineate the concept of preparatory set (PS), referring to the coordination of largely sub-cortical mechanisms that underlie an organism's stress response. Such mechanisms include posture, autonomic state, affective state, attention, and expectation. The authors outline how mind-body approaches such as meditative movement and somatic education can be used to restore an adaptive PS.

### Yoga

The second section of this Research Topic features hypothesis and theory as well as original research articles on yoga. Gard et al. present a theoretical framework and system-based network model outlining the mechanisms through which yoga may impact self-regulation and psychological health. The authors contextualize yoga in historical and contemporary settings, and describe the types of ethical percepts, physical postures, breath regulation, as well as meditative techniques that the practice involves. In addition, they outline how yoga may facilitate bidirectional integration between high- and low-level brain networks, and how this may impact cognitive, emotional, behavioral, and autonomic functioning under stress. Schmalzl et al. also propose a comprehensive theoretical framework of yoga-based practices (YBP), and discuss the main neurophysiological and neurocognitive processes hypothesized to underlie their effects. Specifically, the authors propose that compared to mindfulness-based practices, the rich set of movement, breath, and attention components employed in YBP may more directly engage the vagal afferent system, as well as basal ganglia and cerebellar circuits. In turn, YBP may have a more pronounced impact on autonomic, emotional, and cognitive regulation. Henje Blom et al. present a model for the treatment of adolescent depression informed by mindfulness-based therapy, yoga, as well as modern psychotherapeutic approaches. Their proposed Training for Awareness, Resilience and Action (TARA) takes the developmental limitations of top-down cognitive control in adolescence into account, and promotes bottom-up strategies such as vagal afference to decrease limbic activation and reduce allostatic load. It provides a comprehensive framework for a novel treatment strategy for adolescent depression, and constitutes a base for investigating the neuroscientific and systemic regulatory mechanisms of change in this condition. To conclude the theoretical portion of this section, Solomonova presents an opinion article exploring the concept of yoga as an intentional practice. She outlines the importance of studying intentional and first-person experiential aspects in the neurophenomenological investigation of MECPs, emphasizing various ways in which yoga may shed light on the contribution of intentional and dynamic bodily processes to embodied cognition. As for original research articles, Villemure et al. used magnetic resonance imaging (MRI) to compare age-related gray matter volume (GMV) decline in expert yoga practitioners and controls. Yoga practitioners did not display the typically amount of age-related GMV decline, and years of yoga experience were found to correlate with GMV in brain areas involved in autonomic regulation, emotional processing, and executive functioning. The authors interpret their findings to suggest that sustained yoga practice may have neuroprotective effects against age-related GMV decline. In a further neuroimaging study, Gard et al. compared whole brain resting state functional connectivity in experienced yoga practitioners, meditation practitioners and controls. Network based statistics revealed that, as a group, yoga and meditation practitioners had significantly greater connectivity between the caudate nucleus and numerous cortical regions. The authors conclude that increased functional connectivity within basal ganglia cortico-thalamic feedback loops may be a potential mechanism underlying improved behavioral and cognitive flexibility previously found to be associated with yoga and meditation. Lastly, Fiori et al. present a behavioral study investigating proprioceptive and vestibular processing as well as self-transcendence traits in a group of yoga practitioners and individuals with no yoga experience. The results of their study indicate that yoga practitioners have a higher degree of body awareness characterized by more reliance on internal bodily signals for behavioral regulation. In addition, they point to a potential correlation between body awareness and self-transcendence traits.

### Tai chi

The third section of this Research Topic features three original research articles on tai chi. Wayne et al. report the results of a clinical trial involving a tai chi intervention for older adults. Specifically, the authors investigated the impact of tai chi training on dual task gait parameters that are predictive of falls. The positive effects of tai chi training observed under cognitively challenging conditions support the value of neurophysiological research evaluating how mind-body practices like tai chi impact cognitive-motor interactions. Converse et al. investigated the effect of a tai chi program on self-reported and objectively measured attentional processes in healthy young adults. Outcome measures included self-reported levels of inattention and hyperactivity-impulsivity, as well as performance on a computer based response inhibition task. Both measures were positively impacted by the program, leading the authors to suggest that tai chi may hold potential as a non-pharmacological intervention for ADHD. Lastly, Kerr et al. present two studies evaluating the effect of tai chi on sensorimotor processing. The results of the first study, in which electromyography (EMG) was used to measure intermuscular coherence (IMC) in advanced as well as novice practitioners, suggest that tai chi practice elicits complex changes in sensorimotor processes over the course of training. The findings of the second study indicate that the amount of cumulative practice in tai chi practitioners is related to some aspects of their response to the rubber hand illusion (RHI), an experimental paradigm examining facets of body ownership and agency. Taken together, these studies provide an interesting platform for further investigations of how body-focused contemplative practices impact objective measures of sensorimotor processing and subjective experiences of embodiment.

### Feldenkrais

The fourth section of this Research Topic is dedicated to an original article on the Feldenkrais Method, a movement-based learning method aimed at improving movement organization. Specifically, Verrel et al. used functional magnetic resonance imaging (fMRI) to investigate the short-term neural effects of two subtly different forms of a brief sensorimotor intervention adapted from the Feldenkrais Method. While increased resting state activity in motor areas was observed after both manipulations, their differential pattern suggested that specifically tailored sensorimotor interventions can selectively target lower level sensory areas related to specific body parts or instead engage more broad action related networks.

### Dance

The final section of this Research Topic features an opinion article by van Vugt who explores the notion of classical ballet as a movement-based contemplative practice. Specifically, the author compares ballet to other contemplative practices on the dimensions of cultivation of attention, interoception, meta-cognition, and emotion regulation. Proposed hypotheses for the potentially different neural pathways involved in cultivating contemplation through ballet compared to other contemplative practices are followed by an outline of suggestions for future studies on the topic.

## Conclusion

The current Research Topic aims to address the existing gap in our understanding of the neurophysiological and neurocognitive mechanisms underlying the effects of movement-based contemplative practices such yoga, tai chi, and modern somatic approaches. The featured original research articles report data probing the influence of movement-based contemplative practices on age related GMV decline, functional brain connectivity, sensorimotor processing, multisensory integration, gait parameters, body awareness, and cognitive control. The featured theory articles propose mechanistic models and hypotheses about (1) how movement-based contemplative practices may engage both bottom-up physiological and top-down cognitive processes, and consequently promote autonomic, emotional and cognitive self-regulation, (2) the relationship between motor and mental skills, and (3) the clinical implications of mindful movement. Lastly, the featured perspective articles aim to more clearly define key concepts such as movement, embodiment, contemplation, intention, and meta-cognition as they pertain to movement-based contemplative practices. We trust that the contributions will be of interest to basic scientists, clinical researchers, and contemplative practitioners alike, and hope it will inspire further research in the field.

## Author contributions

LS wrote the manuscript, CEK provided critical feedback, and both authors have approved the work for publication.

## Funding

LS is supported by funding from the U.S. Department of Veteran Affairs Rehabilitation Research & Development (VA RR&D), and a Francisco J. Varela Research Award from the Mind & Life Institute. CEK is supported by the Brown University Contemplative Studies Initiative, the Berkman-Landis Family Foundation Fund, and the Hershey Family Foundation.

### Conflict of interest statement

The authors declare that the research was conducted in the absence of any commercial or financial relationships that could be construed as a potential conflict of interest.

